# How future surgery will benefit from SARS-COV-2-related measures: a SPIGC survey conveying the perspective of Italian surgeons

**DOI:** 10.1007/s13304-023-01613-5

**Published:** 2023-08-14

**Authors:** Leandro Siragusa, Roberta Angelico, Marco Angrisani, Biagio Zampogna, Marco Materazzo, Roberto Sorge, Luca Giordano, Roberto Meniconi, Alessandro Coppola, SPIGC Survey Collaborative Group, Annarita Marino, Giorgio Giraudo, Sofia Esposito, Alessia Urbani, Matteo De Pastena, Rodolfo Mastrapasqua, Mattia Garancini, Alice Frontal, Giovanni Pascal, Jacopo Martellucc, Francesco Falb, Alessandro Boscarelli, Pietro Bertoglio, Eleonora Trecca, Luca Galassi, Vincenzo Vento, Ambra Chiappini, Alessandro Antonelli, Francesco Bennardo, Filippo Familiari, Giuseppe Giannaccare, Andrea Sisto Zappia, Giuseppe Giuliani, Francesca Falcone, Stefano Sebastiani, Mauro Montuori, Stefano Rossi, Andrea Sagnotta, Beatrice Giuliani, Giusy Carmen Imbriani, Stefano Restaino, Lorenzo Andreani, Fabrizio Di Maria, Antonio Simone Laganà, Livio Vitiello, Federico Berton, Edoardo Virgilio, Marco Palisi, Luca Portigliotti, Marco Calussi, Luigi Conti, Claudio Mauriello, Mirko Barone, Edoardo Saladino, Alessia Giaquinta, Domenico Zerb, Giuseppe Frazzetta, Giovanni Merola, Andrea Chierici, Roberto Bini, Leonardo Centonze, Riccardo De Carlis, Luca Ferrario, Alessandro Giani, Andrea Lauterio, Nicolò Tamini, Stefano Corti, Emanuele Botteri, Jacopo Andreuccetti, Rossella D’Alessio, Giovanni Cestaro, Guglielmo Clarizia, Alessandro Spolini, Alberto Salvatore Carboni, Enrico Benzoni, Giampaolo Galiffa, Bruno Perotti, Massimiliano Veroux, Valentina Randazzo, Domenico Topa, Chiara Pranteda, Massimiliano Veroux, Giorgia Contini, Chiara Iacusso, Valerio Voglino, Pietro Vita, Francesco Maria Carrano, Luca Ambrosio, Roberto Cammarata, Gabriella Teresa Capolupo, Damiano Caputo, Filippo Carannante, Chiara Cascone, Francesco Esperto, Tommaso Farolfi, Luca Frasca, Ida Francesca Gallo, Giulia Gibin, Giancarlo Giurazza, Luca Improta, Vincenzo La Vaccara, Paolo Luffarelli, Daniela Luvero, Giovanni Marangi, Gianluca Masciana, Alessandro Mazzola, Erica Mazzotta, Chiara Isabella Miligi, Nunzio Montelione, Antonio Nenna, Paolo Orsaria, Rocco Papalia, Giuseppe Francesco Papalia, Francesco Rosario Parisi, Francesco Prata, Rosa Salzillo, Simone Santini, Francesco Sofo, Andrea Zampoli, Cinzia Tanda, Gaia Altieri, Francesco Ardito, Francesco Belia, Valentina Bianchi, Alberto Biondi, Giuseppe Brisinda, Marco Chiappetta, Gianluca Ciolli, Alessandro Ciolli, Federica Ferracci, Lorenzo Ferri, Valeria Fico, Claudio Fiorillo, Pietro Fransvea, Federica Galiandro, Francesco Giovinazzo, Antonio La Greca, Francesco Litta, Caterina Mele, Donato Paolo Pafundi, Elena Panettieri, Valerio Papa, Romeo Patini, Romeo Patini, Gerardo Perrotta, Caterina Puccioni, Pietro Santocchi, Giulia Armatura, Stefano Olmi, Gianmaria Casoni Pattacini, Stefano Salgarello, Mario Trompetto, Cristina Bombardini, Roberto La Rocca, Giuseppe Celentano, Andrea Micalef, Antonio Mazzella, Alberto Settembrini, Cesare Zoia, Luca Degrate, Giovanbattista Musumeci, Carmen Angela Maria Palopoli, Giulia Montori, Elena Bonati, Vincenza Paola Dinuzzi, Francesco Velluti, Andrea Balla, Davide Edoardo Bonasia, Diego Coletta, Giammauro Berardi, Marco Colasanti, Stefano Ferretti, Camilla Gasparoli, Germano Mariano, Stefano Avenia, Pasquale Cianci, Luca Cestino, Federico Festa, Federico Fazio, Francesca Ascari, Matteo Desio, Gabriela Aracelly Arroyo Murillo, Marco Cereda, Raffaele Galleano, Giulia David, Antonio Pansini, Carlo Gazia, Giulia Atzori, Lorenzo Ferro Desideri, Simone Famularo, Jacopo Galvanin, Veronica Maria Giudici, Giuseppe Mangiameli, Simona Mei, Flavio Milana, Andrea Pansa, Matteo Sacchi, Alberto Testori, Gabriele Di Carlo, Marco Paratore, Umberto Perrone, Aldo Vagge, Jacopo Viganò, Beatrice Torre, Mauro Alessandro Scotti, Gabriele Carbone, Raffaele Cerchione, Paola De Nardi, Lorenzo Gozzini, Luca Ottaviani, Carlotta Senni, Ottavio Piccin, Luca Pio, Francesco Colombo, Riccardo Avantifiori, Valeria Baldassarri, Roberto Caronna, Pierfranco Maria Cicerchia, Diletta Corallino, Daniele Crocetti, Gaetano Gallo, Francesco Giovanardi, Francesca Giovannetti, Redan Hassan, Angelo Iossa, Quirino Lai, Francesco Lancellotti, Alessio Lucarini, Sara Lucchese, Gennaro Mazzarella, Fabio Melandro, Andrea Minervini, Edoardo Maria Muttillo, Livia Palmieri, Rocco Pasqua, Francesco Rosiello, Giacomo Salina, Simone Sibio, Pasqualino Sirignano, Mariarita Tarallo, Sofia Usai, Camilla Vanni, Edoardo Viglietta, Martina Zambon, Nunzia Ivana Conversano, Angelo Gabriele Epifani, Valentina Milano, Luca Sacco, Mariachiara Nava, Anna Maffioli, Simona Giuratrabocchetta, Filippo Baracchi, Michele Zuolo, Marco Ceresoli, Daunia Verdi, Andrea Belli, Francesco Pata, Elisa Piovano, Giovanlorenzo Pastore, Federico Bernabei, Selene Deiana, Alberto Arceri, Claudio D’Agostino, Chiara Marafante, Elisabetta Moggia, Sara Parini, Marco Moretti, Fabio Uggeri, Nicholas Pontarolo, Tommaso Fontana, Graziano Palmisano, Mario Giuffrida, Eleonora Guaitoli, Carlotta Ferretti, Giorgia Iacopino, Rossella Gioco, Giuseppe Roscitano, Paolo Montanelli, Maria Francesca Chiappetta, Enrico Pinotti, Erica Monati, Giada Fazio, Francesco Di Pietro, Francesco Damarco, Andrea Barberis, Andrea Razzore, Angelo Pascale, Sara Loi, Francesco Ferrara, Matteo Rossi, Giorgio Lisi, Giovanni Viel, Diego Sasia, Dario Bono, Emanuele Rampino Cordaro, Elena Giacomelli, Iacopo Giani, Luca Seriau, Gianluca Pellino, Marco Sparavigna, Giuseppe Trigiante, Roberto Giuseppe D’Ambrogio, Francesca Cardella, Sara Guzzetti, Andrea-Pierre Luzzi, Giacomo Carganico, Beatrice Drago, Giancarlo Micheletto, Riccardo Orlandi, Carmen Cutolo, Umberto Gibello, Massimiliano Mistrangelo, Edoardo Forcignanò, Stefano D’Ugo, Pasquale Losurdo, Mattia Manitto, Guido Caroli, Melania Franco, Pier Luigi Tilocca, Paolo Mendogni, Giuseppe Sena, Daniele Sambucci, Claudio Luciani, Pietro Atelli, Agostino Guida, Fabio Marino, Andrea Morini, Maria Grazia Sibilla, Filippo Longo, Sara Giaccari, Vincenzo Vigorita, Alberto Balduzzi, Fabio Barra, Daniele Delogu, Erica Milone, Lapo Bencini, Vittorio Aprile, Piermarco Papini, Nicola Montemurro, Matteo Cavallo, Arcangelo Picciariello, Giovanni Tomasicchio, Alessandra Fittipaldi, Michele Maruccia, Simone Gerardi, Nicola Cillara, Simona Deidda, Giuseppe Demarinis, Enrico Peiretti, Filippo Tatti, Claudio Iovino, Gaetano Isola, Valerio Calogero Progno, Marcello Migliore, Giorgio Badessi, Chiara Barillà, Gaetano Silvio Calleri, Stefano Cianci, Fausto Fama, Francesco Fleres, Carmelo Mazzeo, Mario Gaetano Visaloco, Carlo Marchetto, Federico Bolognesi, Laura Benuzzi, Greta Bracchetti, Francesco Brucchi, Carlo Alberto Manzo, Luca Scaravilli, Carlo Ferrari, Aldo Rocca, Pasquale Napolitano, Pietro Anoldo, Chiara Caricato, Michele Manigrasso, Marco Milone, Luigi Napolitano, Giuseppe Palomba, Vincenzo Schiavone, Martino Vetrella, Ugo Grossi, Lucia Moletta, Alfredo Annicchiarico, Ivan Vella, Giuseppe Talesa, Ugo Boggi, Francesco Aiello, Alessandro Anselmo, Amedeo Antonelli, Giulia Bacchiocchi, Federico Beati, Vittoria Bellato, Federica Billeci, Francesca Blasi, Oreste Claudio Buonomo, Michela Campanelli, Giulia Coco, Alessia Contadini, Luigi Eduardo Conte, Giulia D’Ippolito, Arianna Di Marcantonio, Claudia Fede Spicchiale, Gabriele Gallo Afflitto, Alice Gismondi, Giulio Gorgolini, Alessandra Vittoria Granai, Simona Grande, Andrea Gravina, Andrea Martina Guida, Sara Ingallinella, Laura Keci, Eleonora Latini, Davide Marino, Fabio Massimo Oddi, Luca Orecchia, Cristine Brooke Pathirannehalage Don, Marco Pellicciaro, Lorenzo Petagna, Brunella Maria Pirozzi, Claudia Quaranta, Maurizio Rho, Alessandro Rosina, Maria Sara Santicchia, Federica Saraceno, Alfonso Schiavone, Bruno Sensi, Alessandra Spina, Luca Sullo, Federico Tacconi, Riccardo Tajè, Gianluca Vanni, Danilo Vinci, Giulia Vita, Giuseppe Alba, Simona Badalucco, Ludovico Carbone, Osvaldo Carpineto Samorani, Glauco Chisci, Roberto Cuomo, Alessandro Francia, Daniele Fusario, Bruno Gargiulo, Edoardo Pasqui, Leonardo Pasquetti, Pasquale Puoti, Luca Resca, Jacopo Cumbo, Stefano Ganio, Giuseppe Vizzielli, Marco Anastasi, Domenico Guerra, Andrea Romanzi, Alberto Vannelli, Marco Baia

**Affiliations:** 1 Department of Surgical Sciences, University of Rome “Tor Vergata”, Rome, Italy; 2 HPB and Transplant Unit, Department of Surgical Sciences, University of “Rome Tor Vergata”, Rome, Italy; 3 Department of General Surgery and Liver Transplantation, San Camillo Forlanini Hospital, Rome, Italy; 4 Operative Research Unit of Orthopaedic and Trauma Surgery, Fondazione Policlinico Universitario Campus Bio-Medico, Rome, Italy; 5 Research Unit of Orthopaedic and Trauma Surgery, Department of Medicine and Surgery, Università Campus Bio-Medico di Roma, 00128 Rome, Italy; 6 PhD Program in Applied Medical-Surgical Sciences, Breast Oncoplastic Surgery, University of Rome Tor Vergata, Rome, Italy; 7 Department of Biostatistics, University of Rome Tor Vergata, Rome, Italy; 8 Department of Surgery, Sapienza University of Rome, Rome, Italy

**Keywords:** COVID-19 pandemic, Surgical management, Surgical training, Survey, Trainee, Training program

## Abstract

COVID-19 negatively affected surgical activity, but the potential benefits resulting from adopted measures remain unclear. The aim of this study was to evaluate the change in surgical activity and potential benefit from COVID-19 measures in perspective of Italian surgeons on behalf of SPIGC. A nationwide online survey on surgical practice before, during, and after COVID-19 pandemic was conducted in March–April 2022 (NCT:05323851). Effects of COVID-19 hospital-related measures on surgical patients’ management and personal professional development across surgical specialties were explored. Data on demographics, pre-operative/peri-operative/post-operative management, and professional development were collected. Outcomes were matched with the corresponding volume. Four hundred and seventy-three respondents were included in final analysis across 14 surgical specialties. Since SARS-CoV-2 pandemic, application of telematic consultations (4.1% vs. 21.6%; *p* < 0.0001) and diagnostic evaluations (16.4% vs. 42.2%; *p* < 0.0001) increased. Elective surgical activities significantly reduced and surgeons opted more frequently for conservative management with a possible indication for elective (26.3% vs. 35.7%; *p* < 0.0001) or urgent (20.4% vs. 38.5%; *p* < 0.0001) surgery. All new COVID-related measures are perceived to be maintained in the future. Surgeons’ personal education online increased from 12.6% (pre-COVID) to 86.6% (post-COVID; *p* < 0.0001). Online educational activities are considered a beneficial effect from COVID pandemic (56.4%). COVID-19 had a great impact on surgical specialties, with significant reduction of operation volume. However, some forced changes turned out to be benefits. Isolation measures pushed the use of telemedicine and telemetric devices for outpatient practice and favored communication for educational purposes and surgeon–patient/family communication. From the Italian surgeons’ perspective, COVID-related measures will continue to influence future surgical clinical practice.

## Introduction

The SARS-CoV-2 pandemic has radically changed healthcare systems worldwide. Focusing hospital capacities on the management of a large number of patients with acute respiratory syndrome, a major part of which required intensive care, led to a dramatic decrease of activities among all other clinical services, especially with regard to surgical specialties [1].

Apart from the direct reduction of elective surgical activities during the SARS-CoV-2 pandemic, collateral damage included postponement of disease diagnosis and operation, delays in the oncological pathway, increase in post-operative morbidity, difficulty in outpatient and emergency service access, and also general surgeons’ and patients’ fear of hospital [2–4]. Undoubtedly, the rapid need for resource redistribution and isolation measures to face COVID-19 infection rapidly forced major changes in daily clinical practice.

The SARS-CoV-2 pandemic definitely favored a faster digital transition, the use of alternative operating strategies, and the implementation of personal development tools [5]. Thus, the potential benefit derived from the new measurements adopted since the COVID-19 pandemic across surgical disciplines is still unclear. Through a nationwide survey, we aimed to evaluate the changes in surgical practice and the potential benefit derived from SARS-CoV-2-related measures in the future perspective of Italian surgeons on behalf of the Italian Polyspecialistic Society of Young Surgeons (SPIGC).

## Materials and methods

### Study design

A national online survey on surgical practice before, during, and after the COVID-19 pandemic was conducted in March–April 2022. The survey was designed by a committee of three authors (L.S., M.A., and M.M.), who identified and targeted possible positive evolutions of the SARS-CoV-2 pandemic-driven surgical practice adjustments, and reviewed by four senior authors (A.C., R.A., B.Z., and RM), on behalf of SPIGC. The survey was designed on the SurveyMonkey^©^ web application (SVMK Inc., One Curiosity Way, San Mateo, USA). The purpose of this survey was explained to all participants with a brief introduction. Participants were asked to sign a privacy policy consent form at the start.

The survey was composed of six different sections (S1–S6), including both open and closed questions, as follows: S1 (Q1–Q9), demographic data; S2 (Q10–Q15), pre-operative patient management; S3 (Q16–Q23), peri-operative management; S4 (Q24–Q27), post-operative management; S5 (Q28–Q30), professional development; S6 (Q31–Q32), professional volume. Questions in S2–S5 related to pre-COVID, COVID, and post-COVID periods; and those in S6 (personal activity volume) to pre-COVID and COVID periods. In addition, a final section comprising feedback, with a rating scale of 1–5 (Q33), and a box for comments (Q34) was included. The survey structure is summarized in Table 1.

**Table 1:** Survey structure

S1 Demographics	
Q1 Generalities (full name, email, affiliation, ORCID)	
Q2 Age	
Q3 Gender	
Q4 Italian region of practice	
Q5 Surgical specialty	
Q6 Personal role	
Q7 Type of hospital	
Q8 Hospital role during pandemic	
Q9 Type of referral center	
S2 Pre-operative management (pre-COVID, COVID, post-COVID)	
Q10 Percentage of telematic outpatient consultations?	
Q11Percentage of laboratory and radiological exams evaluated telematically?	
Q12 Percentage of patients undergoing pre-admission tests externally?	
Q13 Percentage of patients requiring admission before the operation for pre-admission tests?	
Q14 Percentage of patients managed with conservative treatment in an elective regimen?	
Q15 Percentage of patients managed with conservative treatment in urgency?	
S3 Peri-operative management (pre-COVID, COVID, post-COVID)	
Q16 Percentage of operation doable in outpatient setting performed without admission?	
Q17 Percentage of operation doable as day-surgery performed with admission less than 24 h?	
Q18 Percentage of operation doable with locoregional anesthesia technique performed with this modality?	
Q19 Percentage of PPE use to avoid in-hospital infections?	
Q20 Percentage of patients following fast-track protocols?	
Q21 Percentage of patient–family discussions performed telematically	
Q22 Percentage of elective national health system practices performed in other accredited structures?	
Q23 Percentage of operations performed in a private setting	
S4 Post-operative management (pre-COVID, COVID, post-COVID)	
Q24 Percentage of post-operative follow-up consultations performed telematically?	
Q25 Percentage of patients in which telemetric devices were used for post-operative home care?	
Q26 Percentage of patients communicating directly through private channels?	
Q27 Percentage of multidisciplinary meetings online?	
S5 Personal development (pre-COVID, COVID, post-COVID)	
Q28 Percentage of online participation to educational events?	
Q29 Percentage of time dedicated to training with a simulator out of total time used for professional development?	
Q30 Percentage of time dedicated to online surgery videos out of total time used for professional development?	
S6 Volume	
Q31 Monthly pre-COVID	Number of outpatient and follow-up consultations; laboratory and radiological tests evaluated; patients pre-admitted for operation (in election, in urgency), ambulatory or day-surgery; multidisciplinary meetings and educational events attended; operation videos watched; hours dedicated to professional development
Q32 Monthly COVID	
Q33 Feedback	
Q34 Comments	

All questions from Q10 to Q30 are reported before COVID-19 pandemic (pre-COVID), during COVID-19 pandemic (COVID), and after COVID-19 pandemic (post-COVID)

The pre-COVID period was defined as professional surgical practice until March 2020; the COVID time frame is from March 2020 until the end of the pandemic, a future period in which social distancing and personal protective equipment (PPE) will be no longer be required and restrictions ended; and the post-COVID period starts after the above-mentioned scenario.

Italian surgeons from any surgical specialty were considered eligible for the survey’s analysis. Any type of surgical trainee and junior and senior surgeons were included. All participants were informed that the results of the survey would be used for further statistical evaluation and scientific publication. Anonymity was guaranteed by the study design and authorship in the collaborative group was offered on a voluntary base.

A dedicated account to deliver the survey was created: surveyspigc@gmail.com. This was used to distribute the survey, answer query-related inconsistencies in survey responses, and confirm the final authorship. The survey was promoted through a mailing list of Italian surgeons, instant message services (WhatsApp, Telegram), the official SPIGC site/account, and social media (Facebook, Instagram and Linked-in).

A pilot version of the survey was tested with 17 participants from 7 to 14 March 2022 and, after approval of all committee authors, the revised final version was launched on 15 March 2022 and closed on 30 April 2022.

From 1 to 15 May 2022, the authors of incomplete answers were personally contacted through email to complete the survey. Only those who completed all sections of the survey were considered in the final data analysis. On 15 May 2022, the full dataset was extracted from Survey Monkey, incomplete responses and duplicates were deleted, and the final database was sent to the statistician for result analysis.

### Outcome measures

The main outcome of the study was to evaluate, over time, the effect of COVID-19 hospital-related measures on pre-, peri-, and post-operative management of patients and personal surgical development from all surgical specialties, exploring the potential benefits of future surgery. The question outcome was matched with the corresponding activity volume when applicable.

### Ethics

Survey participation was voluntary and no incentives were offered. According to the Institutional Review Board of the Policlinico Tor Vergata, no approval was required. This survey was registered at clinicaltrials.gov. NCT05323851.

Reporting of this study follows the American Association for Public Opinion Research reporting guidelines and the CHEcklist for Reporting Results of Internet E-Surveys (CHERRIES) [6].

### Statistical analysis

Datasets were collected in Excel (Microsoft, Redmond, WA, USA) and the statistical analysis was performed using SPSS (IBM SPSS Statistics for Windows, Version 27.0; IBM Corp., Armonk, NY, USA). Characteristics were summarized by means of levels for categorical variables or quantiles for continuous variables. All tests were two sided, accepting *p* < 0.05 as indicating a statistically significant difference, and confidence intervals were calculated at the 95% level.

## Results

### Study population

Of 581 surgeons who answered the survey, 108 respondents were excluded (105 incomplete, 3 duplicates) and 473 complete responses were included for analysis (completion rate 82%). Participants had a mean age of 35.3 ± 7.7 years and males accounted for 73.2% of respondents (*n* = 346). Based on the region of employment in Italy, surgeons were distributed as follows: 42.8% in Central regions (*n* = 202), 26.6% in Northwest regions (*n* = 126), 11.4% in Southern regions (*n* = 54), 10.6% in Northeast regions (*n* = 50), and 8.6% in Island regions (*n* = 41).

According to the surgical specialty, respondents included: 66.8% (*n* = 316) general surgeons, 6.1% (*n* = 29) orthopedic surgeons, 4.7% (*n* = 22) thoracic surgeons, 4.7% (*n* = 22) vascular surgeons, 4.3% (*n* = 20) oculists, 2.7% (*n* = 13) gynecologists, 2.7% (*n* = 13) dental surgeons, 2.1% (*n* = 10) urologists, 1.7% (*n* = 8) plastic surgeons, 1.7% (*n* = 8) pediatric surgeons, 1.1% (*n* = 5) otorhinolaryngology surgeons, 0.6% (*n* = 3) maxillary surgeons, 0.4% (*n* = 2) heart surgeons, and 0.4% (*n* = 2) neurosurgeons.

Participants were mainly consultants (42.9%, *n* = 203), followed by surgical trainees (28.8%, *n* = 136), PhD surgeons (13.5%, *n* = 64), assistant professors (9.9%, *n* = 47), and professors (4.9%, *n* = 23).

Respondents’ institutions included academic hospitals (45.9%, *n* = 217), public hospitals (30.7%, *n* = 145), research centers (18%, *n* = 85), and private hospitals (5.5%, *n* = 26). During the pandemic the respondents’ institutions changed, with 82.8% (*n* = 392) in mixed hospitals (COVID and non-COVID services), 10.6% (*n* = 50) exclusively in COVID centers, and 6.6% (*n* = 31) in non-COVID centers. Participants’ referral hospitals were secondary centers (53.1%, *n* = 251), tertiary centers (30.9%, *n* = 146), and peripheral centers (16%, *n* = 76).

**Fig. 1:**
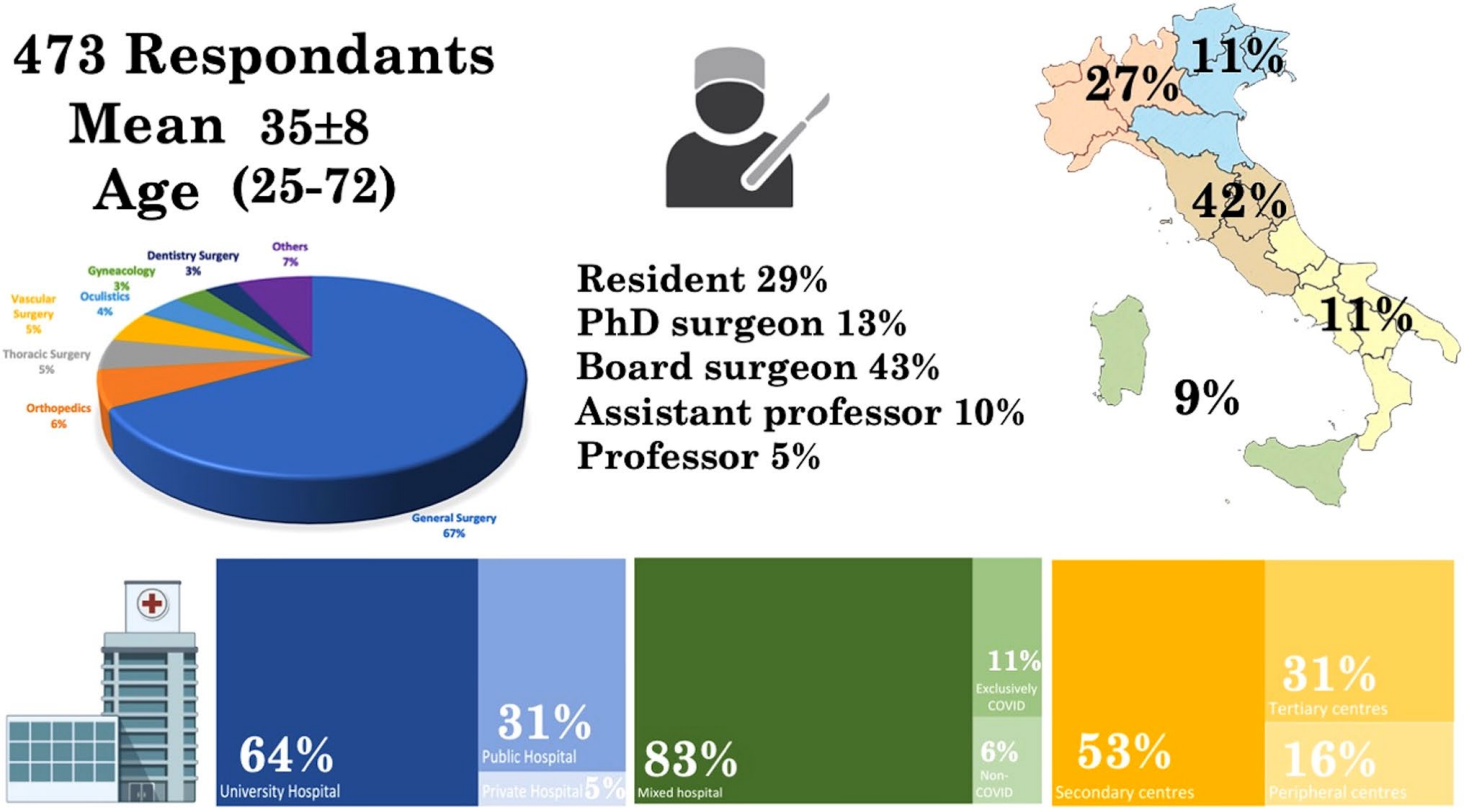
Survey respondents demographics

The demographic data are summarized in Fig. 1 and Table 2.

**Table 2:** Demographic characteristics

	Study population (*n* = 473)
Q2 Age (Mean ± SD, range) (years)	35.3 ± 7.7 (25–72)
Q3 Sex Ratio (M:F)	127:346 (26.8%:73.2%)
Q4 Italian region of practice	North-West 126 26.6%
	North-East 50 10.6%
	Center 202 42.8%
	South 54 11.4%
	Islands 41 8.6%
Q5 Surgical specialty	General surgery 316 66.8%
	Orthopedics 29 6.1%
	Thoracic surgery 4.7% 22
	Vascular surgery 4.7% 22
	Oculistics 20 4.3%
	Gynecology 13 2.7%
	Dentistry surgery 13 2.7%
	Urology 10 2.1%
	Plastic surgery 8 1.7%
	Pediatric surgery 8 1.7%
	ENT 51.1%
	Maxillae surgery 3 0.6%
	Hearth surgery 2 0.4%
	Neurosurgery 2 0.4%
Q6 Personal role	Resident 136 28.8%
	PhD surgeon 64 13.5%
	Board certificate surgeon 203 42.9%
	Assistant Professor 47 9.9%
	Professor 23 4.9%
Q7 Type of hospital	Public Hospital 145 30.7%
	Private Hospital 26 5.5%
	University Hospital 302 63.9%
Q8 Hospital role during pandemics	Non-COVID center 31 6.6%
	Mixed hospital 392 82.8%
	Exclusively COVID center 50 10.6%
Q9 Type of referral center	Peripheral centers 76 16%
	Secondary centers 251 53.1%
	Tertiary centers 146 30.9%

Values are *n* (%) unless otherwise indicated

### Pre-operative management

The management of patients’ candidate to surgical operations in the pre-COVID, COVID, and post-COVID periods is reported in Table 3. Before the COVID-19 pandemic, the mean number of patient consultations as outpatients was 90.1 ± 116 per month, which decreased to 29.7 ± 43 during the COVID period (*p* < 0.0001). Since the pandemic, the use of telematic consultations (4.1% vs. 21.6%; *p* < 0.0001) and telematic diagnostic evaluations (16.4% vs. 42.2%; *p* < 0.0001) significantly increased compared to the pre-COVID period. Additionally, surgeons perceived that higher adoption of both the virtual consultation and telematic diagnostic evaluation will be maintained also in the post-COVID period (13.9% and 30.9% of cases, respectively).

**Table 3:** Pre-operative management

	Pre-Covis	Covis	Post-Covis	*P*-value
Outpatient clinic patient volume/month(mean ± SD)	90.1 ± 116	29.7 ± 43	–	0.0001
Q10 Telematic outpatient consultation	4.1%	21.6%	13.9%	Pre-Cov–Cov 0.0001 Cov–Post-Cov 0.0001 Pre-Cov–Post-Cov 0.0001
Diagnostic test volume/month (mean ± SD)	91 ± 465	28.3 ± 41	–	0.0001
Q11 Telematic tests evaluation	16.4%	42.2%	30.9%	Pre-Cov–Cov 0.0001 Cov–Post-Cov 0.0001 Pre-Cov–Post-Cov 0.0001
Pre-admission patient volume/month (mean ± SD)	57 ± 91	23.1 ± 40	–	0.0001
Q12 External pre-admission tests	15.6%	25.9%	21.4%	Pre-Cov–Cov 0.0001 Cov–Post-Cov 0.0001 Pre-Cov–Post-Cov 0.0001
Q13 Need of admission for pre-admission tests	17.8%	24.6%	17.8%	Pre-Cov–Cov 0.0001 Cov–Post-Cov 0.0001 Pre-Cov–Post-Cov 0.811
Elective patient volume/month (mean ± SD)	27.7 ± 60	9.9 ± 14	–	0.0001
Q14 Conservative management in elective regimen	26.3%	35.7%	28.4%	Pre-Cov–Cov 0.0001 Cov–Post-Cov 0.0001 Pre-Cov–Post-Cov 0.0001
Urgency patient volume/month (mean ± SD)	7.1 ± 12	6.1 ± 10	–	0.028
Q15 Conservative management in urgency regimen	20.4%	38.5%	24.1%	Pre-Cov–Cov 0.0001 Cov–Post-Cov 0.0001 Pre-Cov–Post-Cov 0.0001

Values are *n* (%) unless otherwise indicated

Pre-operative tests were performed externally to the treating hospital in 15.6% of cases in the pre-COVID period and in 25.9% of cases during the COVID pandemic (*p* < 0.0001); these figures are predicted to remain unchanged in the future (21.4%). The percentage of patients needing hospital admission for pre-operative tests was 17.8% in the pre-COVID period, 24.6% during COVID, and is predicted to be 17.8% post-COVID (*p* < 0.0001).

For all surgical specialties, elective surgical activities significantly reduced from the pre-COVID to the COVID period (surgical procedures per month: 27.7 ± 60.2 vs. 9.9 ± 14; *p* < 0.0001). Moreover, compared to the pre-COVID phase, during the COVID period surgeons opted more frequently for conservative management in patients, with a possible indication for elective surgery (26.3% vs. 35.7%); this approach was predicted to be maintained in 28.4% of cases post-COVID (*p* < 0.0001).

The mean number of urgent operations per month was 7.1 ± 12 pre-COVID, which decreased to 6.1 ± 10 during the COVID period (*p* = 0.028). Also, conservative management of patients with a possible indication for urgent surgery increased from 20.4% pre-COVID to 38.5% during COVID, with a prediction of 24.1% post-COVID (*p* < 0.0001).

### Peri-operative management

During the pre-COVID period, surgical procedures listed in public healthcare hospitals were performed in other accredited structures in 11.9% of cases; this modality was adopted in 21.3% of operations during COVID, with a prediction of 17.8% post-COVID (*p* < 0.0001). Also, the percentage of operations performed in a private setting remained stable in the pre-COVID and COVID periods (15.9% vs. 14.2%). Thus, participants believe that the percentage of patients operated on in the private setting will increase after the COVID period (20%; *p* < 0.0001).

According to the regimen of admission for surgical procedures, participants stated that operations performed both in outpatient settings (48.7% vs. 27.5%; *p* < 0.0001) and in a one-day (< 24 h) admission regimen (53.9% vs. 29.2%; *p* < 0.0001) significantly reduced from pre-COVID to the COVID period. Responders believe that in the post-COVID period, operations that can be performed as an outpatient or on one-day admission will be used in 46.2% and 51.1% of cases, respectively. Moreover, the percentage of operations possible with locoregional anesthesia was 55% during pre-COVID, 46% during COVID and is predicted to be 57% post-COVID (*p* < 0.0001).

Peri-operative fast-track protocols (i.e., Enhanced Recovery After Surgery [ERAS]) were adopted in 42.9% of operations during pre-COVID and this percentage decreased to 41.9% during COVID; thus, responders predict that they will be used in more than half of cases (51.5%) post-COVID (*p* < 0.0001).

To avoid in-hospital infections, the use of PPE increased significantly during the COVID pandemic (27.9% vs. 94.3%; *p* < 0.0001). Interestingly, participants stated that the adoption of PPE would be maintained also in the future (80.7%).

Telematic consultation with patients’ family was more frequently adopted during the COVID pandemic (13.2% vs. 76%) and responders believe that this figure will be maintained in almost 48.7% of cases (*p* < 0.0001).

The peri-operative management data are summarized in Table 4.

**Table 4:** Peri-operative management

	Pre-Covis	Covis	Post-Covis	*P*-value
Outpatient surgery patient volume/month (mean ± SD)	13 ± 35	2.8 ± 8	–	0.0001
Q16 Outpatient surgery setting (%)	48.7%	27.5%	46.2%	Pre-Cov–Cov 0.0001 Cov–Post-Cov 0.0001 Pre-Cov–Post-Cov 0.001
Day surgery patient volume/month (mean ± SD)	11.3 ± 18	2.8 ± 6	–	0.0001
Q17 Day surgery setting (%)	53.9%	29.2%	51.1%	Pre-Cov–Cov 0.0001 Cov–Post-Cov 0.0001 Pre-Cov–Post-Cov 0.001
Q18 Loco-regional anesthesia operation (%)	55%	46%	57%	Pre-Cov–Cov 0.0001 Cov–Post-Cov 0.0001 Pre-Cov–Post-Cov 0.001
Q19 PPE use (%)	27.9%	94.3%	80.7%	Pre-Cov–Cov 0.0001 Cov–Post-Cov 0.0001 Pre-Cov–Post-Cov 0.0001
Ward patient managed/month (mean ± SD)	36.4 ± 35	20 ± 22	–	0.0001
Q20 Fast-track protocols application (%)	42.9%	41.9%	51.5%	Pre-Cov–Cov 0.343 Cov–Post-Cov 0.0001 Pre-Cov–Post-Cov 0.0001
Q21 Telematic patient family discussion (%)	13.2%	76%	48.7%	Pre-Cov–Cov 0.0001 Cov–Post-Cov 0.0001 Pre-Cov–Post-Cov 0.0001
Q22 elective SSN practice performed in other accredited structures (%)	11.9%	21.3%	17.8%	Pre-Cov–Cov 0.0001 Cov–Post-Cov 0.0001 Pre-Cov–Post-Cov 0.0001
Q23 Private setting operation (%)	15.9%	14.2%	20.2%	Pre-Cov–Cov 0.064 Cov–Post-Cov 0.0001 Pre-Cov–Post-Cov 0.0001

### Post-operative management

Results on post-operative management are detailed in Table 5. Although the mean number of patients visited monthly in the post-operative outpatient clinic decreased during the COVID period (36.5 ± 40 vs. 17 ± 2 3 in the pre-COVID and COVID periods, respectively; *p* < 0.0001), the percentage of post-operative follow-up consultations performed telematically increased from 5.6% to 26% during COVID (*p* < 0.0001). Moreover, respondents feel that post-operative telematic consultations will be adopted in 15.6% of cases in the post-COVID period (*p* < 0.0001).

**Table 5:** Post-operative management

	Pre-Covis	Covis	Post-Covis	*P*-value
Post-operative follow-up patient/month (mean ± SD)	36.5 ± 40	17 ± 23	–	0.0001
Q24 Telematic post-operative follow-up consultation (%)	5.6%	26%	15.6%	Pre-Cov–Cov 0.0001 Cov–Post-Cov 0.0001 Pre-Cov–Post-Cov 0.0001
Q25 Telemetric device use for post-operative home care (%)	3.8%	14%	11.1%	Pre-Cov–Cov 0.0001 Cov–Post-Cov 0.0001 Pre-Cov–Post-Cov 0.0001
Q26 Direct patient contact through private channels (%)	23.9%	47.5%	38.3%	Pre-Cov–Cov 0.0001 Cov–Post-Cov 0.0001 Pre-Cov–Post-Cov 0.0001
MDT participation/month (mean ± SD)	4.3 ± 4	4.4 ± 7	–	0.811
Q27 Percentage of MDT meeting online (%)	7.9%	79.4%	52.3%	Pre-Cov–Cov 0.0001 Cov–Post-Cov 0.0001 Pre-Cov–Post-Cov 0.0001

The use of telemetric devices for post-operative home care has increased significantly since the COVID pandemic (3.8% vs. 14%; *p* < 0.0001) and surgeons believe that they will continue to be used in the future (11.1%).

Post-operatively, communication with patients was performed more frequently through private channels (personal emails, telephone calls and messages) adopted during the COVID phase compared to the pre-COVID period (23.9% vs. 47.5%; *p* < 0.0001), with a prediction of 38.3% post-COVID (*p* < 0.0001).

The percentage of online multidisciplinary meetings increased from 7.9% pre-COVID to 79.4% during COVID (*p* < 0.0001), and surgeons believe that telematic meetings will be maintained in more than half of cases (52.3%) in the future.

### Surgeons’ personal development

Pre-COVID, the mean number of educational events participated in was 2.2 ± 3 per month for each participant, which increased to 4.7 ± 6 per month during COVID (*p* < 0.0001). Online courses (i.e., webinars, seminars, classes) increased from 12.6% to 86.6% during the pre-COVID and COVID periods, respectively, and surgeons perceive that in the post-COVID phase such online educational events will be maintained for more than half (56.4%) of cases.

Surgeons dedicated a mean time of 28.4 ± 46 h per month to their professional development during pre-COVID and this time increased to 34.3 ± 51 h during the COVID period (*p* < 0.0001).

Also, participants declared that they used a surgical training simulator for about 10.6% of their time before COVID, which increased to 17.1% during COVID (*p* < 0.0001) and is predicted to be 18.3% in the post-COVID period.

The percentage of time dedicated to online surgery videos used for professional development increased from 10.0% to 29.1% in the pre-COVID and COVID periods, respectively (*p* < 0.0001), and surgeons believe that they will maintain to consult online surgical videos in the future. The personal development data are summarized in Table 6.

**Table 6:** Professional development

	Pre-Covis	Covis	Post-Covis	*P*-value
Educational event participation/month (mean ± SD)	2.2 ± 3	4.7 ± 6	–	0.0001
Q28 Online educational event participation (%)	12.6%	86.6%	56.4%	Pre-Cov–Cov 0.0001 Cov–Post-Cov 0.0001 Pre-Cov–Post-Cov 0.0001
Hours dedicated to professional development/month (mean ± SD)	28.4 ± 46	34.3 ± 51	–	0.0001
Q29 Professional development time dedicated to simulator training (%)	10.6%	17.1%	18.3%	Pre-Cov–Cov 0.0001 Cov–Post-Cov 0.194 Pre-Cov–Post-Cov 0.0001
Q30 Professional development time dedicated to surgical video e-Learning (%)	10.9%	29.1%	21%	Pre-Cov–Cov 0.0001 Cov–Post-Cov 0.0001 Pre-Cov–Post-Cov 0.0001

**Fig. 2:**
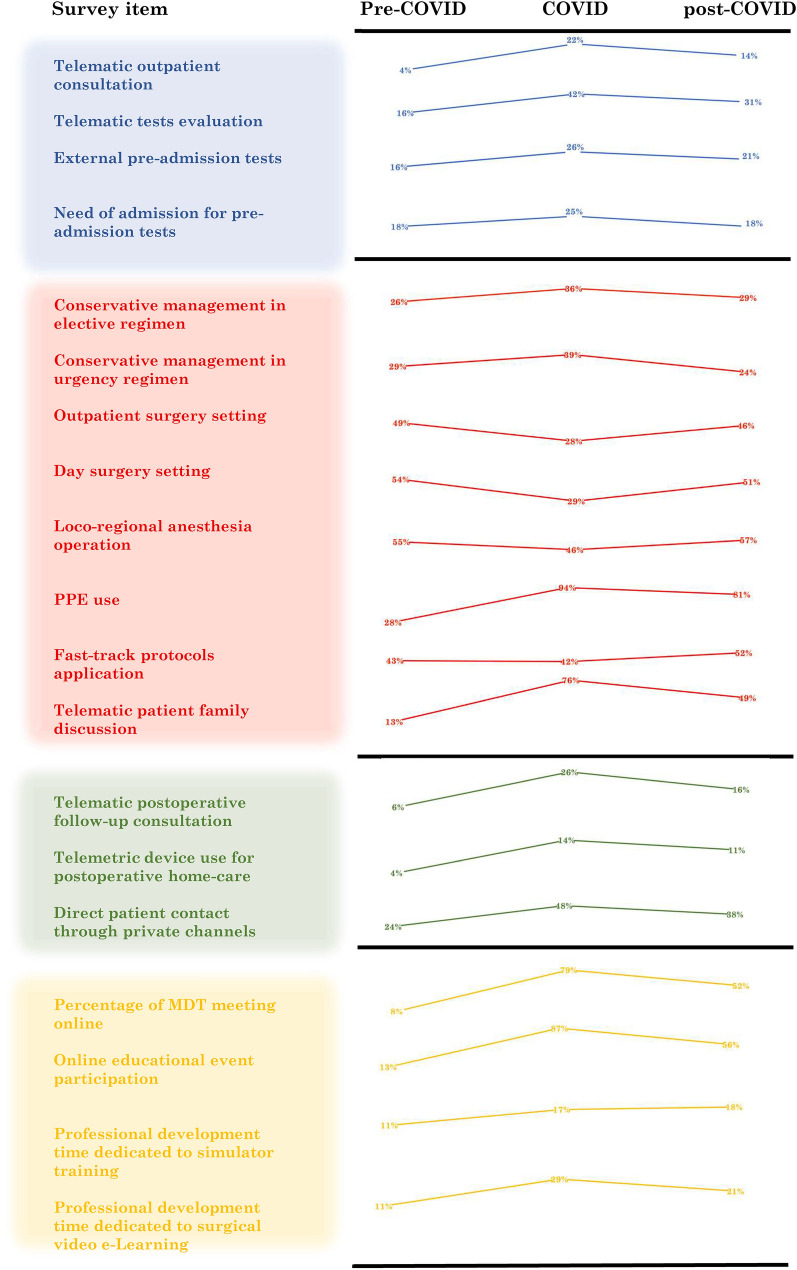
Survey outcomes summary

All survey outcomes are summarized in Fig. 2

## Discussion

This survey investigated surgical practice in Italy before, during, and “what-is-expected-to-be” after the SARS-CoV-2 pandemic. While clear time frames were established for the pre-COVID and COVID periods, the survey design did not initially include a specific start date for the post-COVID period. However, it is now possible to retrospectively designate May 2023 as the start for the post-COVID period, since declared by the WHO as the end of the pandemic [7]. Substantial differences were identified between the pre-COVID and COVID periods; moreover, while some of the introduced novelties are expected to vanish in the post-pandemic phase, it appears that others might have to stay.

There are numerous previous studies that reported several negative effects of the pandemic (including increased comorbidity, delay in diagnosis, etc.), but very few have addressed the positive transitions that it ushered in [8, 9]. The scope of this study was to identify possible appreciated modifications that could ultimately be adopted and change future practice after the COVID pandemic in the clinical management of surgical patients across different specialties and in the personal education of surgeons.

After the SARS-CoV-2 pandemic, in surgical practice, one major change introduced was in outpatient management. During the COVID period, there was a marked reduction in patient and diagnostic test ambulatory evaluation, which was replaced by online consultation and remote diagnostic test evaluation. Although this practice was already relatively diffuse in some countries (i.e., UK), it was extremely limited in others, such as Italy. Indeed, after the COVID-19 pandemic, the Italian healthcare system’s long-term plan aims to make digital consultation the mainstream source of outpatient management by 2024 [10]. Surgeons involved in the survey are convinced that this practice will remain, probably because it permits optimization of resources and time, patient comfort and attendance rate. Furthermore, remote consulting switches have already been demonstrated to mitigate stress and in-hospital infection risk, especially among frail patients (elderly, immunosuppressed) [11–15].

In a similar way, the patient’s physical presence at the hospital was limited as much as possible, even during necessary steps for elective surgery such as pre-hospitalization. In fact, the percentage of patients who had their pre-operative work-up elsewhere (usually closer to home) increased. This practice is thought to decrease centralization for routine diagnosis, unburdening major hospitals and favoring an improved territorial distribution. Similarly, decentralization occurred for benign and/or uncomplicated diseases, with private practice increasing. The following measures were implemented to cope with increased waiting list times that unfortunately lead, over the pandemic period, to serious delay, also in oncology patients, with a proven negative impact on patients’ health [2, 16–18]. All these changes are thought to persist and may help to enhance the overall efficiency of the national health system. Decentralization was reported also in the higher rate of surgical procedures performed in private and private-accredited structures.

In the same direction is the predicted implementation on a greater scale of ERAS protocols and locoregional anesthesia: the pandemic necessities might have helped reluctant surgeons to appreciate the advantages of these approaches, despite there being no great change in these items during the COVID-19 period [19, 20]. This experience may have built trust and confidence in the effectiveness of ERAS, leading to its continued and increased adoption in the future, despite shortage of resources and workforce may hider its systematic use. Surprisingly, other measures such as one-day or ambulatory surgery decreased during the pandemic, as already demonstrated for specific surgery such as laparoscopic cholecystectomy [21]. This may be due to reshaped in-hospital logistics, but the absence of a predicted increase in the future also suggests a possible general weak belief in this surgical practice.

Therapeutic management of surgical disease was significantly different among these time frames. During the pandemic, conservative management increased drastically both in the elective and emergency setting [5, 22–25]. Surgeons involved in the survey believe that this trend might continue, albeit at a lower level, as the conservative strategy has proved useful in many cases. It is possible that the pandemic has revived conservative management in situations that were considered indications for surgery, with acceptable outcomes. This might also have increased surgeons’ sensitivity to indicate surgery in cases with the greatest expected benefit. The use of PPE, of course, increased dramatically with COVID-19 and surgeons think that this trend will also stay, as PPE may prove helpful in reducing nosocomial infections [4, 26, 27].

Telemetric measures for post-operative follow-up have also been introduced and are now very popular among survey respondents. These measures include online consultation platforms, hospital-provided tablets for questionnaire filling, dedicated chats with hospital personnel and vital sign detection using smart-watches [28, 29]. Online/telephonic measures were used increasingly also for surgeon–patient and especially for surgeon–patient/family contact, with a dramatic 15–80% rise. This may have broken the Italian taboo of physical presence being necessary for correct communication. Again, all these technologies may reduce the workload for surgeons and increase patient satisfaction and outcomes [30].

Online peer-to-peer contact has also increased, from multidisciplinary meetings to events such as e-congresses and educational surgical videos. This may speed the process of professional development, and probably also reduce costs, and was indeed a welcomed innovation [31–33]. E-congresses are easy to sign up for and swift to follow. Surgical videos have democratically given everyone the opportunity to learn from the best surgeons around the world. Similarly, the time spent on simulator training has almost doubled and investment in this form of training may help trainees to speed up their learning curve. In fact, all these measures are predicted to continue to increase in the near future.

This study has several limitations. First, the survey methodology is intrinsically biased in relation to question phrasing and respondents’ subjectivity. Second, the sample, although conspicuous, is mostly composed of general surgeons and, thus, may not represent the real picture of all specialties. Finally, the future predicted values are based on the perspective of the Italian surgeons and may be inaccurate. Thus, this study has highlighted the positive effects of the SARS-CoV-2 crisis on Italian surgical practice. In particular, it appears that the pandemic has boosted the implementation of technology in patient management, both pre- and postoperatively, and in surgical professional development.

The SARS-CoV-2 pandemic had a great impact on surgical specialties, with a significant reduction of patient and operation volume but with some forced changes that turned out to be benefits. The isolation measures pushed the use of telemedicine and telemetric devices for outpatient practice and favored communication for educational purposes and surgeon–patient/family communication. Different clinical and organizational strategies were utilized in patient management to reallocate surgical volume and improve patient outcomes. In the Italian surgeons’ perspective, this change of course driven by COVID-19 will continue and develop further in future clinical practice.

## Data Availability

Anonymized interview is available on request to the working group and upon reasonable request to the corresponding author.
